# Changes in Social, Romantic, and General Life Satisfaction Over the Course of a Substance Use Disorder

**DOI:** 10.3389/fpsyt.2021.734352

**Published:** 2021-10-28

**Authors:** Nina C. Christie, Vanya Vojvodic, Pranav Meda, John R. Monterosso

**Affiliations:** ^1^Department of Psychology, University of Southern California, Los Angeles, CA, United States; ^2^Department of Preventive Medicine, Keck School of Medicine, University of Southern California, Los Angeles, CA, United States; ^3^Keck School of Medicine, University of Southern California, Los Angeles, CA, United States; ^4^Department of Psychology, College of Life Sciences, University of California, Los Angeles, Los Angeles, CA, United States; ^5^Brain and Creativity Institute, University of Southern California, Los Angeles, CA, United States; ^6^Neuroscience Graduate Program, University of Southern California, Los Angeles, CA, United States

**Keywords:** social connection, life satisfaction, social satisfaction, quality of life, opioid, substance use, recovery, prescription opioid

## Abstract

**Background:** The pandemic has highlighted the importance of social connection for health and well-being. Satisfaction across domains of life is associated with substance use outcomes, such as risk of relapse and mortality. Previous work has delineated the relationship between substance use and social connections, yet there is a lack of research exploring the relationship between substance use and satisfaction with domains of life over time.

**Methods:** We retrospectively assessed satisfaction with social life, romantic life, and general life across five phases of substance use among 339 adults, of whom 289 identify as formerly having a problem with substance use, and a comparison group of 50 who report no history of problematic drug use. We compared those whose primary drug of choice was alcohol, marijuana, methamphetamine, non-prescription opioids, and prescription opioids.

**Results:** Those who used prescription opioids reported a larger drop in satisfaction in social life, romantic life, and general life during the course of substance use than those who used other drugs. However, we report no significant differences in *current* satisfaction, social well-being, or quality of life between people in recovery and people with no history of problematic substance use.

**Conclusions:** These findings—alongside neuropsychological work on the opioid system and sociality—paint a picture that those who formerly used prescription opioids may experience lower satisfaction across life domains during the course of their substance use than those who used other substances. However, people in prolonged recovery—regardless of their drug of choice—all show similar levels of satisfaction compared to people with no history of problematic substance use.

## Introduction

The widespread impacts of the COVID-19 pandemic on social relationships have highlighted their importance for our psychological and physical well-being (1, 2). Humans are hyper-social beings (3), and our success as a species is strongly linked to our capacity for flexible cooperation (4). As individuals, our health, longevity, and happiness all appear to be linked to the maintenance of strong social bonds (5, 6). Higher degrees of social connection are associated with lower risks of inflammation and physiological dysfunction (6). However, in recent years, there has been a downward trend in the number of close personal relationships people maintain (7, 8).

This decline in personal relationships is concerning given that high quality friendships are positively correlated with overall life satisfaction (9), which is in turn associated with lower risk of mortality (10). Those who feel more lonely or isolated have lower life satisfaction as a result of feeling a need to belong, relative to those who are less isolated (11). Social capital (trust in relationships, belonging to a group, and socializing) is highly correlated with overall life satisfaction across dozens of countries (12).

Additionally, recent work has shown that community-level social capital is negatively correlated with per capita fatal drug overdoses (13). During the pandemic, adults in the United States who report higher levels of social isolation had (a) lower life satisfaction across domains, and (b) were more likely to use substances to cope with life stressors (14). Other studies have documented a significant increase in both the initiation and maintenance of substance use during the pandemic, including a large spike in opioid overdose deaths (15–17), that has coincided with markedly increased feelings of isolation brought on by social distancing measures designed to reduce the spread of COVID-19 (1, 2, 18). This evidence points to the idea that isolation and satisfaction (e.g., social, romantic, and general) are associated with substance use behaviors.

Substance use is a well-documented coping mechanism for social distress. The Diagnostic and Statistical Manual of Mental Disorders (19) explicitly outlines social impairment as part of the diagnostic criteria for substance use disorders. Risk factors for relapse often include social components, such as a lack of social support from friends or family, or poor quality of social relationships (20). Interventions aim to target the decline of social relationships that often accompanies substance use disorders by providing new social connections within a recovery community (21), by focusing on repairing damaged relationships within the individual's community (22), or by encouraging social involvement to combat the powerful reinforcing properties of drug use (23). Peer-run treatment modalities for substance use disorders routinely focus on human connection through shared experiences of addiction, including peer support groups like Alcoholics Anonymous. A recent Cochrane review reported that Alcoholics Anonymous and other 12-step facilitation programs are as effective as other established treatments, such as cognitive behavioral therapy (CBT), and even outperform CBT in prolonged abstinence (24).

In addition to diagnostic criteria and treatment regimens, neural correlates illuminate the relationship between social relationships and substance use disorders—specifically opioid use disorder. The brain opioid theory of social attachment (BOTSA) outlines the critical role of the endogenous opioid system in social attachment (25). The endogenous opioid system plays a key role in the reward associated with social ties across the life span of humans and non-human primates, including (a) maternal/infant bonds, (b) non-kin relationships, and (c) romantic relationships. For more details, please see the 2011 paper by Machin and Dunbar. It is important to note that humans consume pharmacologically similar drugs in different social contexts: prescription and non-prescription opioids are not consumed in the same way, by the same people, nor in the same places. There is evidence that those who use solely heroin (rather than prescription opioids) are more socioeconomically disadvantaged, older, and more disconnected from social institutions, while those who use solely prescription opioids (rather than heroin) are more likely to be economically stable, connected to social institutions, and less likely to have a history of criminal justice involvement (26). Those who use prescription opioids are also at risk for developing a later heroin use disorder, particularly those who initiate pharmaceutical opioid misuse at a younger age and use it exclusively to get high, rather than those who are introduced to it via the medical system (27). Additionally, among those who are prescribed opioids by a physician, those who have a college degree are 2.5 times more likely to develop an opioid use disorder than those who do not (28). We hypothesized that those who used opioids would show differential satisfaction with their social, romantic, and/or general lives throughout the course of substance use because of the unique pharmacological association between opioids and interpersonal connections as described by BOTSA. However, we separately evaluated those who used prescription and non-prescription opioids because of the differences in sociodemographic characteristics and experiences of stigma between these groups. These differences may be associated with divergent outcomes (e.g., life satisfaction, social wellbeing). As high-risk opioid use remains a prominent issue, further exploration of the relationship between substance use (particularly opioid use) and satisfaction with interpersonal connections is warranted.

While the link between substance use and social isolation has been established, there is a lack of evidence exploring the relationship between substance use and a person's satisfaction with their social, romantic, and general life over the course of time. There is also a dearth of information regarding how prescription opioid use may differ from non-prescription opioid use in terms of social, romantic, and general life satisfaction. In the present study, we aim to retrospectively assess changes in satisfaction in a person's social, romantic, and general life circumstances among those with a history of problematic substance use, and assess whether any differences persist during recovery. We also contemporaneously measure social well-being and quality of life. We sought to address three main questions: (1) Is satisfaction in life domains *pre-drug use* different for people who used different substances? (2) Is satisfaction in life domains predicted by an interaction between a person's former drug of choice and time (i.e., *during different stages throughout the course of substance use)*? (3) Is satisfaction in life domains, social well-being, and quality of life *in recovery* different for people who used different substances and people with no history of problem drug use?

## Method

### Sample Selection

The current study employed a retrospective survey design to assess those with a history of problem substance use alongside a comparison sample of participants with no history of problem substance use. The present study used a convenience sample; the sampling universe consisted of adults who reside in the United States and who have an account with Prolific Academic Ltd., an online data collection platform (Prolifiic.co) that has good transparency, functionality, and a relatively high minimum hourly payment for participants (29). Prolific allows researchers to select participants using a two-part survey in which participants complete a screening survey and are subsequently invited to complete the full study. All participants completed the full study between June 6th, 2021 and August 14th, 2021. We used the Drug Abuse Screening Test (DAST-10; a validated clinical measure) to identify participants with a history of at least a low level of problems related to drug use—defined as scoring a three or above on the scale (30, 31). Previous literature has found that a cutoff score of three or higher is associated with a DSM-3R diagnosis of substance abuse or substance dependence (32). A literature review of effective behavioral health screening tools within primary care settings supported the use of the DAST-10 with a cutoff score of three or higher for a substance use disorder, with a discussion of using two as a lower cutoff score for primary care applications (33). We posted a screening survey on Prolific to identify individuals who met our inclusion criteria: individuals screened into the full study if they had (a) a history of problems associated with drug use (i.e., scored a 3 or above on the DAST-10), (b) currently do not engage in problematic substance use (i.e., are not using any substances, or use occasionally/casually with no problems), and (c) were adults currently residing in the United States who have Prolific accounts. Individuals were asked to identify their primary drug of choice in the screening survey. We were interested in comparing those who had a history of problematic use of prescription opioids, non-prescription opioids, methamphetamine, marijuana, and alcohol to one another. We asked about substances other than opioids because methamphetamine, marijuana, and alcohol are also widely used substances and provide controls for opioids among a population of people who use substances. We oversampled those who used opioids and methamphetamine to ensure that we had an adequate sample size to statistically compare between groups. We oversampled by selectively inviting those who indicated that their primary drug of choice was opioids or methamphetamine in the screener to complete the full survey until we had ~50 participants in each category for drug of choice. Additionally, we collected responses from 50 individuals who reported *no* former or current problem with substance use to serve as a comparison group. The control participants did not complete the DAST-10 as they previously indicated that they had never experienced problematic substance use.

### Measures

The full survey for all participants (including controls) who screened in included the following: Social Well-being Scale (34); Quality of Life 35-item measure (35); and demographic questions on age, ethnicity, socioeconomic status. People with a history of substance use also completed the 8-item Inventory of Drug Taking Situations (36) and substance use questions about their age of first intoxication (from any substance, not only drug of choice) and time in sobriety/recovery.

Lastly, there were several measures in which participants with a history of substance use were instructed to answer the same set of questions repeatedly, but each time focusing on a different period of their substance use history: (1) before the initiation of substance use, (2) in the initial stages, (3) in the height/midst of substance use, (4) in the initial stages of reducing or quitting substance use, and (5) in recovery from substance use (either abstinence or non-problematic use). Questions were binned into these 5 blocks, each one representing a phase of substance use. Assessment of satisfaction across life domains were single-item Likert-style questions. Participants were asked the following questions in each block: (a) how satisfied are/were you with your social life, (b) how satisfied were/are you with your romantic life, (c) how satisfied were/are you with your overall life, and (d) were social relationships more/less/similarly desirable compared to [the previous stage]. Control participants were asked these same four questions, but only for the contemporaneous or current time (e.g., how satisfied are you with your social/romantic/general life?). All study measures were approved by the local Institutional Review Board. All statistical analyses were completed using R version 1.1.4.

### Analytic Procedures

#### Demographic Differences

We utilized chi-square tests to determine if there were significant differences between groups in demographic characteristics (e.g., education) and measures of drug use (e.g., substance use severity score).

#### Satisfaction Prior to Substance Use

We used ANOVA to predict pre-drug use satisfaction separately for each domain: social, romantic, and general life satisfaction based on the participant's reported primary substance of choice. For each of the three models, we included participant age, DAST score (i.e., substance use severity), and age of first intoxication as covariates, and the reference group was people who reported alcohol as their primary substance of choice. We did not assess reliability metrics for any of the satisfaction measures as they were single-item measures that retrospectively assessed satisfaction across the 5-stages of substance use.

#### Satisfaction During the Course of Substance Use

For our primary analyses, we used ANOVA to predict satisfaction across the three domains (social, romantic, and general life) based on the interaction between *time* (the 5 stages of substance use assessed in the current study—before initiation, initial stages, height of problematic use, initial cessation, and recovery/current time) and substance of choice, with alcohol as the reference group. In addition, we performed a secondary analysis examining these same variables as change scores relative to satisfaction prior to drug use in a two-step approach: first without removing the variance linked to drug use severity and age, and then a second time after removing the variance associated with drug use severity and age. These secondary analyses using change scores and residual change scores are included in the Appendix.

#### Current Satisfaction and Well-Being (Post-problem Use)

We used ANOVA to predict current satisfaction across the three life domains by drug of choice with the comparison group (i.e., participants with no history of drug use) as the reference. Covariates in the model were current age, age of first intoxication, and DAST.

In addition to questions about current satisfaction with life domains, participants completed the Social Well-being Scale and the Quality of Life Scale. We created two models for each scale: in the first model we used ANOVA to predict separately (a) social well-being and (b) quality of life by drug of choice, including the comparison group (no history of drug use) as the reference and age as a covariate. For the second model, we only included individuals with a history of substance use problems, and used ANOVA to predict current social well-being by the interaction between drug of choice (with alcohol as the reference group), and time in sobriety (<1 year as the reference group), including age and drug use severity as covariates.

## Results

### Demographic Characteristics

Our sample consists of 339 adults (178 males). Among the 339 participants, 50 were comparison participants, and 289 participants reported a history of problems with substance use. See Table 1 for demographic characteristics of the current sample, and see Table 2 for the characteristics of drug use among the sample.

**Table 1:** Demographic characteristics of the full sample including control participants (*N* = 339) by substance of choice.

**Basic descriptive statistics**	**Control**	**Alcohol**	**Marijuana**	**Methamphetamine**	**Prescription opioids**	**Non-prescription opioids**	***P*-value**
	***N =* 50**	***N =* 103**	***N =* 58**	***N =* 32**	***N =* 48**	***N =* 48**	
**Age**							0.081
Median (IQR)	36 (29–46)	33 (29–39)	30 (25–42)	39 (33–47)	36 (30–42)	32 (28–41)	
Missing	0 (0%)	0 (0%)	1 (2%)	0 (0%)	1 (2%)	1 (2%)	
**Income**							0.062
< $10,000	3 (6%)	6 (6%)	5 (9%)	3 (9%)	2 (4%)	5 (10%)	
$10,000–$29,999	8 (16%)	23 (22%)	7 (12%)	8 (25%)	8 (17%)	9 (19%)	
$30,000–$49,999	6 (12%)	30 (29%)	18 (31%)	10 (31%)	10 (21%)	9 (19%)	
$50,000–$79,999	18 (36%)	17 (17%)	14 (24%)	6 (19%)	15 (31%)	14 (29%)	
$80,000–$99,999	6 (12%)	12 (12%)	6 (10%)	2 (6%)	4 (8%)	4 (8%)	
$100,000 or more	9 (18%)	15 (15%)	7 (12%)	3 (9%)	8 (17%)	6 (12%)	
Missing	0 (0%)	0 (0%)	1 (2%)	0 (0%)	1 (2%)	1 (2%)	
**Race**							**0.045**
American Indian or Alaska Native	0 (0%)	1 (1%)	0 (0%)	0 (0%)	0 (0%)	0 (0%)	
Asian	8 (16%)	8 (8%)	6 (10%)	1 (3%)	1 (2%)	0 (0%)	
Black or African American	8 (16%)	10 (10%)	5 (9%)	1 (3%)	1 (2%)	3 (6%)	
Hispanic	1 (2%)	8 (8%)	2 (3%)	2 (6%)	0 (0%)	0 (0%)	
Multiethnic	5 (10%)	8 (8%)	5 (9%)	3 (9%)	5 (10%)	6 (12%)	
White	28 (56%)	68 (66%)	39 (67%)	25 (78%)	40 (83%)	38 (79%)	
Missing	0 (0%)	0 (0%)	1 (2%)	0 (0%)	1 (2%)	1 (2%)	
**Education**							**<0.0001**
Less than high school	0 (0%)	0 (0%)	0 (0%)	0 (0%)	1 (2%)	1 (2%)	
High school graduate	5 (10%)	14 (14%)	7 (12%)	7 (22%)	6 (12%)	8 (17%)	
Some college but no degree	8 (16%)	24 (23%)	9 (16%)	15 (47%)	18 (38%)	14 (29%)	
Associate degree	4 (8%)	4 (4%)	7 (12%)	7 (22%)	5 (10%)	7 (15%)	
Bachelor's degree	19 (38%)	41 (40%)	28 (48%)	2 (6%)	11 (23%)	14 (29%)	
Master's degree	7 (14%)	19 (18%)	6 (10%)	0 (0%)	5 (10%)	3 (6%)	
Doctoral degree	0 (0%)	0 (0%)	0 (0%)	1 (3%)	1 (2%)	0 (0%)	
Professional degree	7 (14%)	1 (1%)	0 (0%)	0 (0%)	0 (0%)	0 (0%)	
Missing	0 (0%)	0 (0%)	1 (2%)	0 (0%)	1 (2%)	1 (2%)	
**Employment**							**0.041**
Employed	29 (58%)	57 (55%)	36 (62%)	10 (31%)	21 (44%)	24 (50%)	
Self-Employed	4 (8%)	10 (10%)	6 (10%)	9 (28%)	12 (25%)	10 (21%)	
Not working (disabled)	0 (0%)	6 (6%)	2 (3%)	6 (19%)	3 (6%)	3 (6%)	
Unemployed	13 (26%)	26 (25%)	11 (19%)	6 (19%)	11 (23%)	10 (21%)	
Retired	3 (6%)	2 (2%)	1 (2%)	1 (3%)	0 (0%)	0 (0%)	
Missing	1 (2%)	2 (2%)	2 (3%)	0 (0%)	1 (2%)	1 (2%)	

*The far-right column is the p-value associated with chi-squared analyses to detect differences between participants for each demographic characteristic*.

*The bold values indicate a significant difference on that demographic characteristic between groups with different primary drugs of choice*.

**Table 2:** Substance use history and characteristics of the subsample of participants with a history of problematic substance use (*N* = 289).

**Basic descriptive statistics**	**Alcohol**	**Marijuana**	**Methamphetamine**	**Prescription opioids**	**Non-prescription opioids**	***P*-value**
	***N =* 103**	***N =* 58**	***N =* 32**	***N =* 48**	***N =* 48**	
Substance use severity	6 (4–7)	5 (4–7)	7 (6–8)	7 (5–8)	7 (5–9)	**<0.0001**
Time in Sobriety						0.096
<1year	33 (32%)	18 (31%)	7 (22%)	6 (12%)	11 (23%)	
1–5 Years	51 (50%)	28 (48%)	13 (41%)	25 (52%)	22 (46%)	
6+ years	19 (18%)	12 (21%)	12 (38%)	17 (35%)	15 (31%)	
Missing	0 (0%)	0 (0%)	0 (0%)	0 (0%)	0 (0%)	
Age of First Intoxication	18 (16–19)	16 (14–18)	15 (14–16)	16 (14–18)	16 (14–18)	**0.010**

*The far-right column is the p-value associated with chi-squared analyses to detect differences between participants for each substance use characteristic*.

*The bold values indicate a significant difference on that substance use characteristic between groups with different primary drugs of choice*.

### DAST-10

Participants completed the DAST-10 measure as a retrospective evaluation of problems they had *ever* experienced due to their substance use (i.e., not a current measure of severity of problems associated with substance use). Higher DAST-10 scores were observed among those whose drug of choice was prescription opioids (*N* = 48, B = 0.87, *t* = 2.82, *p* < 0.01), non-prescription opioids (*N* = 48, B = 1.28, *t* = 4.17, *p* < 0.001), or methamphetamine (*N* = 32, B = 1.26, *t* = 3.54, *p* < 0.001) relative to those whose drug of choice was alcohol (*N* = 103).

### Pre-drug Use

#### Social Life Satisfaction

Prior to the initiation of substance use, those who used marijuana reported lower satisfaction in their social lives relative to those who used alcohol (B = −0.69, *t* = −2.41, *p* < 0.05). Higher age is also associated with higher pre-use social satisfaction (B = 0.02, *t* = 2.08, *p* < 0.05). There was no significant association between severity of substance use and social life satisfaction prior to substance use.

#### Romantic Life Satisfaction

Prior to the initiation of substance use, those who used prescription opioids reported higher satisfaction in their romantic lives relative to those who used alcohol (B = 0.88, *t* = 2.64, *p* < 0.01). There was no significant association with either age or severity of substance use.

#### General Life Satisfaction

Prior to the initiation of substance use, those who used prescription opioids reported higher satisfaction in their social lives relative to those who used alcohol (B = 0.76, *t* = 2.61, *p* < 0.01). Higher age was also associated with higher pre-use general life satisfaction (B = 0.02, *t* = 2.53, *p* < 0.05).

### During the Course of Substance Use

#### Social Life Satisfaction

**Figure 1:**
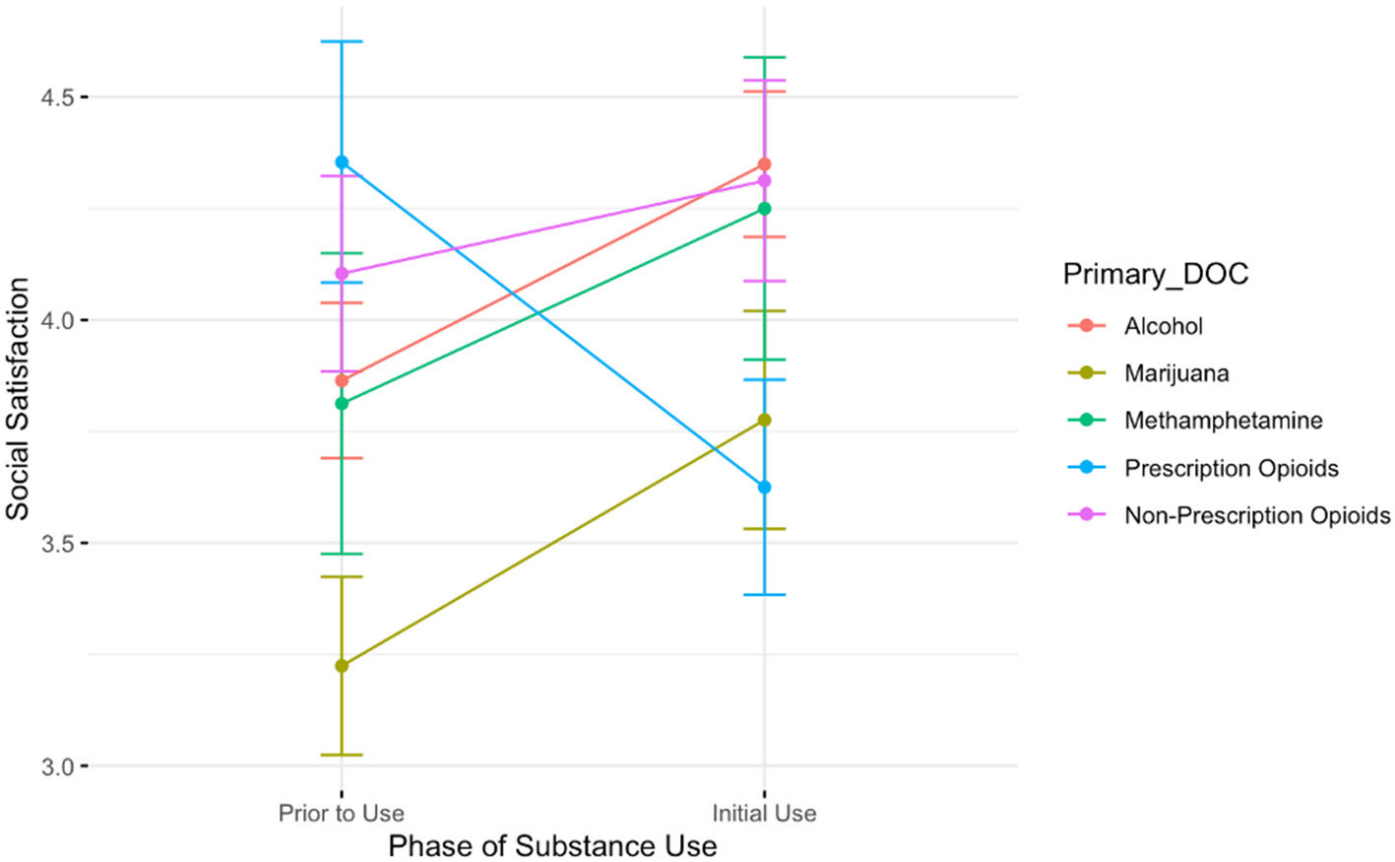
A plot showing the average social satisfaction scores for participants prior to the onset of substance use and during the initial stages of use. Lines are colored by participant primary substance of choice. The only group showing a decrease in social satisfaction is those who report using prescription opioids.

The ANOVA evaluating the relationship between drug of choice and the time from pre-substance use to initial stages of use on feelings of social life satisfaction revealed a significant main effect of time and drug of choice; satisfaction tended to be higher in the initial use phase than before the initiation of use (B = 0.49, *t* = 2.03, *p* < 0.05), and those who used marijuana reported lower satisfaction than those who used alcohol (B = −0.64, *t* = −2.27, *p* < 0.05). Figure 1 depicts the significant interaction; those who used prescription opioids were the only participants who reported a *decrease* in social life satisfaction from pre-substance use levels to the initial stages of use (B = −1.21, *t* = −2.86, *p* < 0.005).

**Figure 2:**
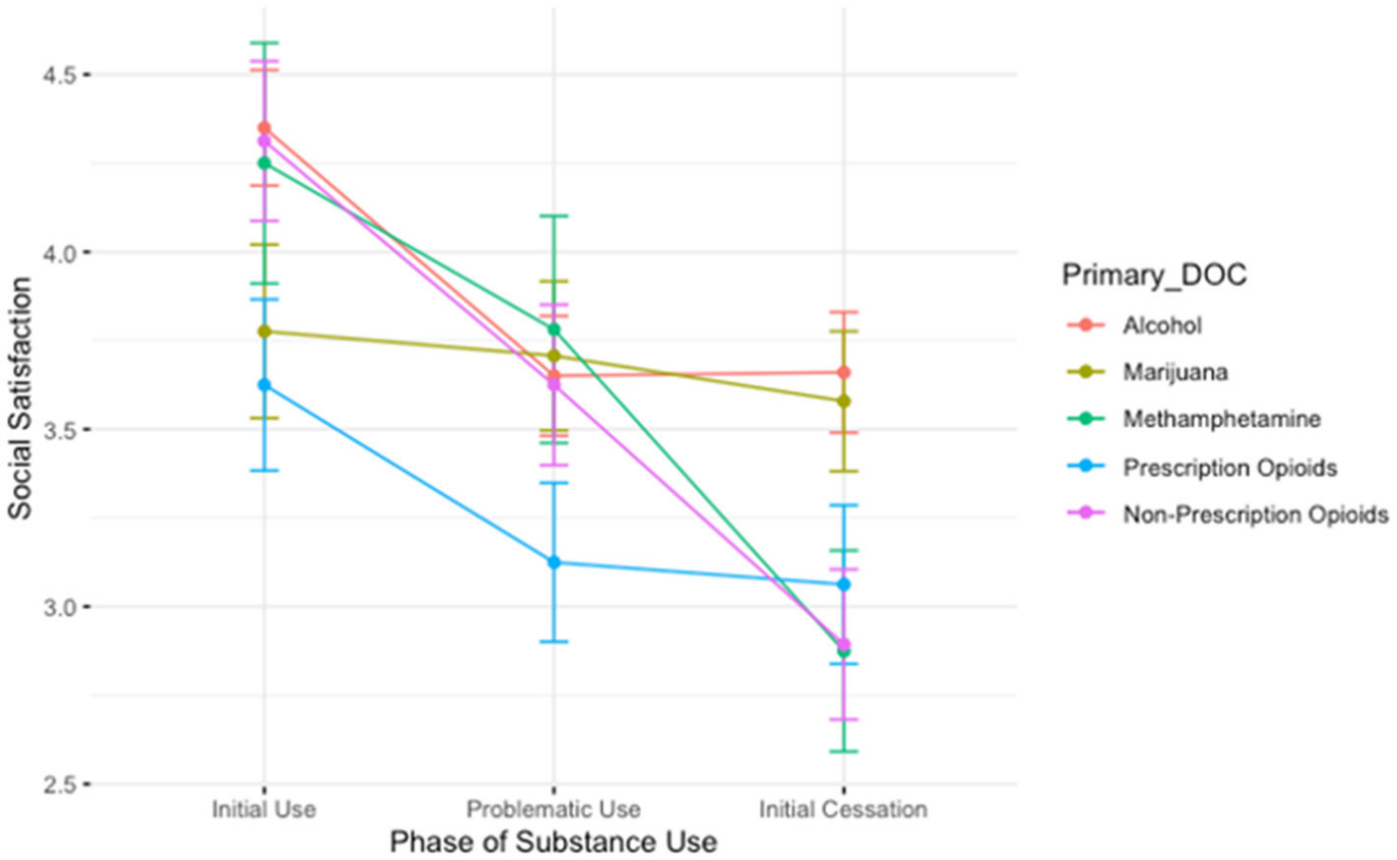
A plot showing the average social satisfaction scores for participants during the initial stages of use, the height of problematic use, and during the initial stages of cessation. Lines are colored by participant primary substance of choice. The groups showing the steepest decrease in social satisfaction between the height of use and period of initial cessation are those who reported using methamphetamine and non-prescription opioids, while those who used prescription opioids reported lower satisfaction than those in other groups during the height of problematic use.

The association between drug of choice and time is depicted in Figure 2. The three time-points *during* use (initial use, height of use, and initial cessation) revealed a main effect of time: relative to the initial phases of substance use, people report lower feelings of social satisfaction both during the height of problematic use (B = −0.70, *t* = −3.03, *p* < 0.01) and during the initial stages of quitting/reducing use (B = −0.69, *t* = −2.99, *p* < 0.01). We also observed a main effect of drug of choice: relative to alcohol, people report lower feelings of social satisfaction if their primary drug of choice was either marijuana (B = −0.57, *t* = −2.11, *p* < 0.05) or prescription opioids (B = −0.72, *t* = −2.51, *p* < 0.05). There was no significant interaction between drug of choice and time.

#### Romantic Life Satisfaction

**Figure 3:**
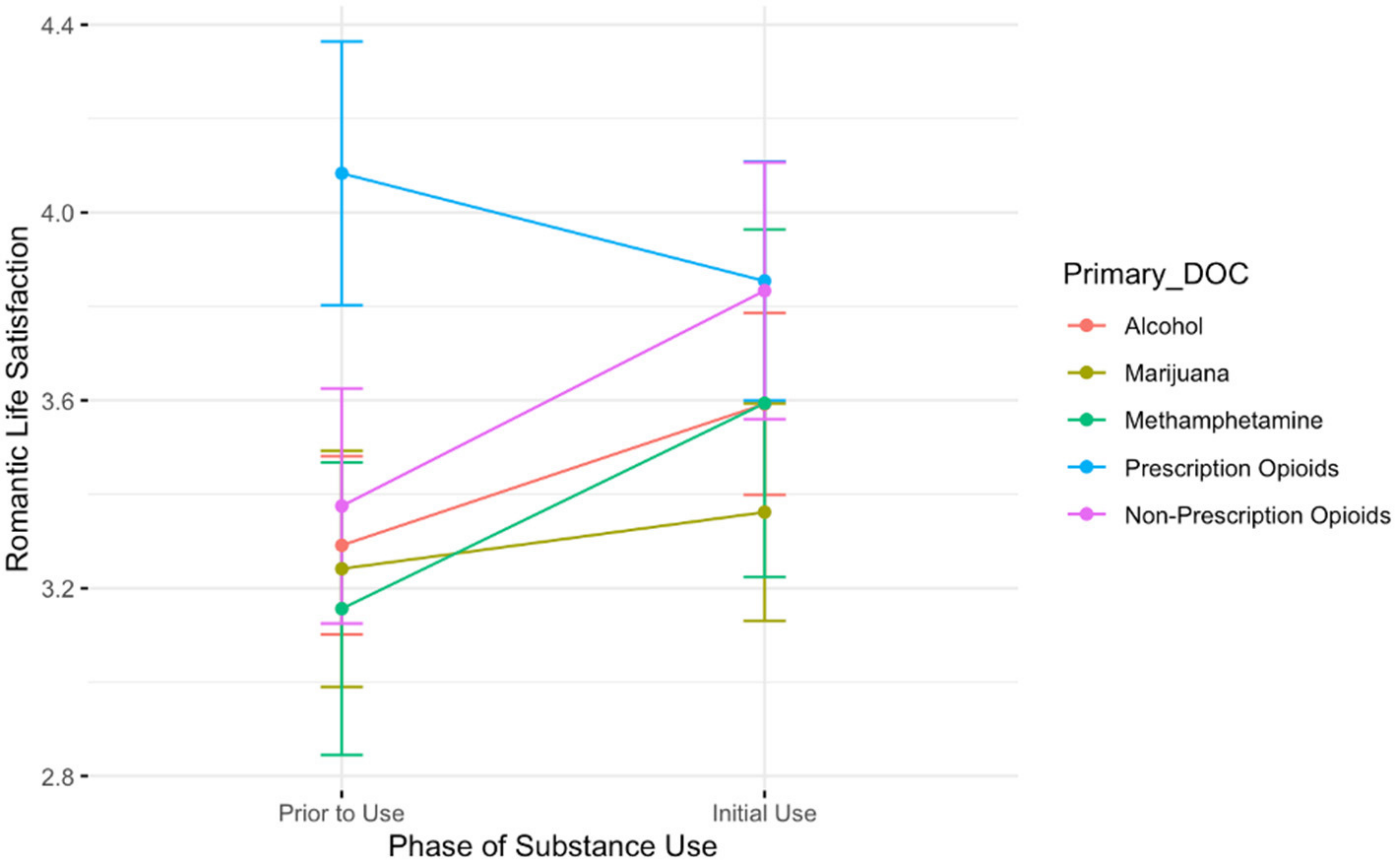
A plot showing the average romantic life satisfaction scores for participants prior to the onset of substance use and during the initial stages of use. Lines are colored by participant primary substance of choice. The only group showing a decrease in romantic life satisfaction between these two timepoints is those who reported using prescription opioids.

The ANOVA evaluating the association between drug of choice and the time from pre-substance use to initial stages of use on feelings of romantic life satisfaction is depicted in Figure 3. It revealed a significant main effect of drug of choice; romantic life satisfaction is higher among those who used prescription opioids relative to those who used alcohol (B = 0.79, *t* = 2.40, *p* < 0.05). There was no significant interaction between time and drug of choice. Again, the only group that shows a decline in romantic satisfaction pre-substance use to the initial stages is the group of those with a history of prescription opioid use.

**Figure 4:**
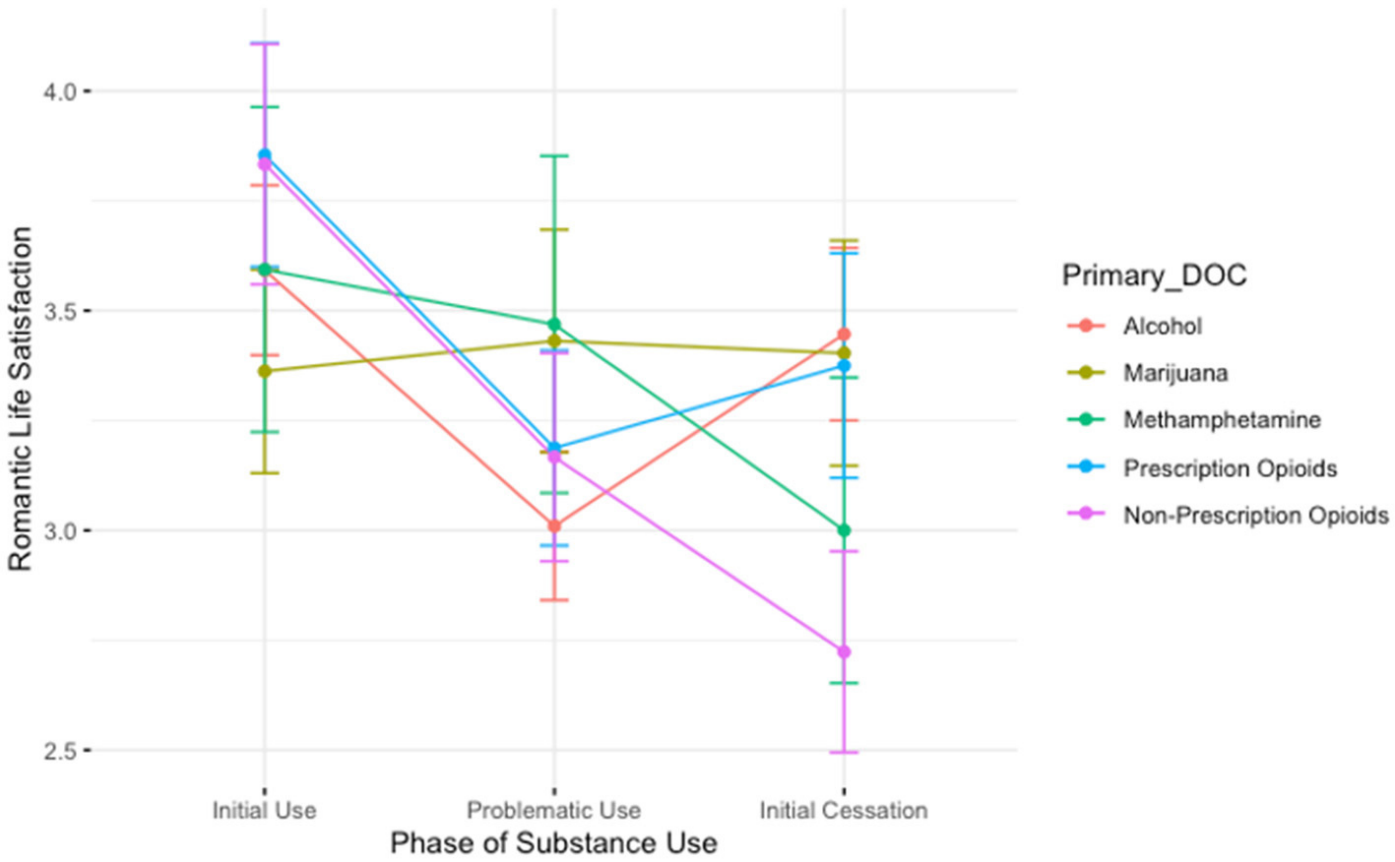
A plot showing the average romantic satisfaction scores for participants during the initial stages of use, the height of problematic use, and during the initial stages of cessation. Lines are colored by participant primary substance of choice. Those who use non-prescription opioids report lower satisfaction than those in other groups during the initial period of cessation. We also generally see a “V”-shaped pattern among those who used alcohol and prescription opioids, where romantic satisfaction is lowest in the height of problematic use. However, for those who used methamphetamine and non-prescription opioids, romantic satisfaction continues to decrease from the height of problematic use to the initial cessation period.

The ANOVA evaluating the association between drug of choice and time for the three time-points *during* use (initial use, height of use, and initial cessation) revealed a main effect of time: relative to the initial phases of substance use, people report lower feelings of romantic life satisfaction during the height of problematic use (B = −0.58, *t* = −2.26, *p* < 0.05). This finding is depicted in Figure 4. We observed no main effect of drug of choice. However, there was a significant interaction between drug of choice and time: those who used non-prescription opioids reported lower romantic life satisfaction during the initial cessation period than those who used alcohol (B = −0.96, *t* = −2.10, *p* < 0.05).

#### General Life Satisfaction

**Figure 5:**
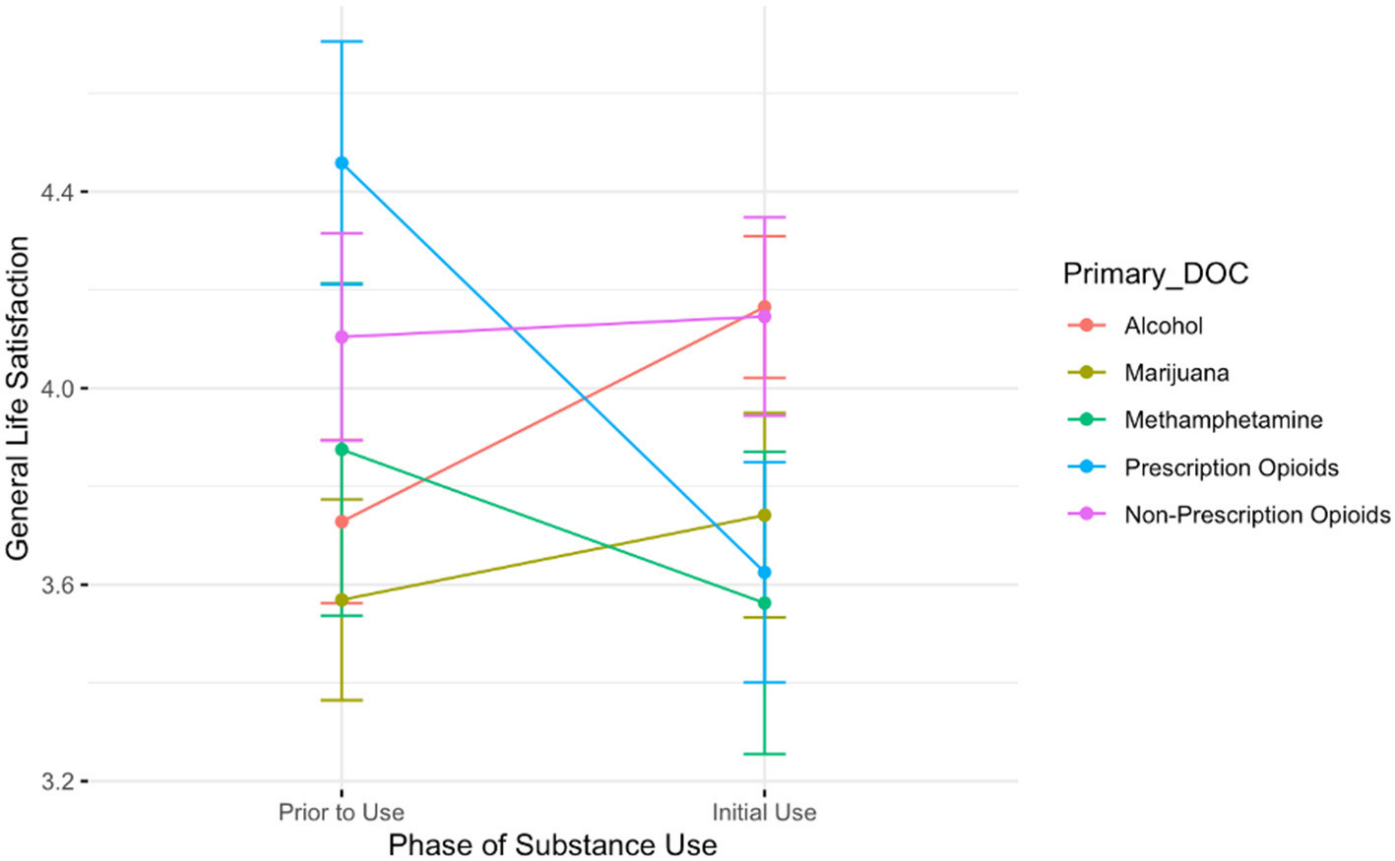
A plot showing the average general life satisfaction scores for participants prior to the onset of substance use and during the initial stages of use. Lines are colored by participant primary substance of choice. The group showing the steepest decrease in general life satisfaction between these two timepoints is those who reported using prescription opioids.

Figure 5 depicts the interaction between drug of choice and the time from pre-substance use to initial stages of use on feelings of general life satisfaction. This analysis revealed a significant main effect of time; participants report higher satisfaction overall before the initiation of drug use (B = 3.73, *t* = 23.76, *p* < 0.001). Additionally, there was a main effect of drug, such that those who used prescription opioids reported higher satisfaction in general than those who used alcohol (B = 0.73, *t*^.^= 2.63, *p* < 0.01). There was also a significant interaction; those who used prescription opioids reported *lower* general life satisfaction than those who use alcohol in the initial stages of use (B = −1.27, *t* = −3.23, *p* < 0.01).

**Figure 6:**
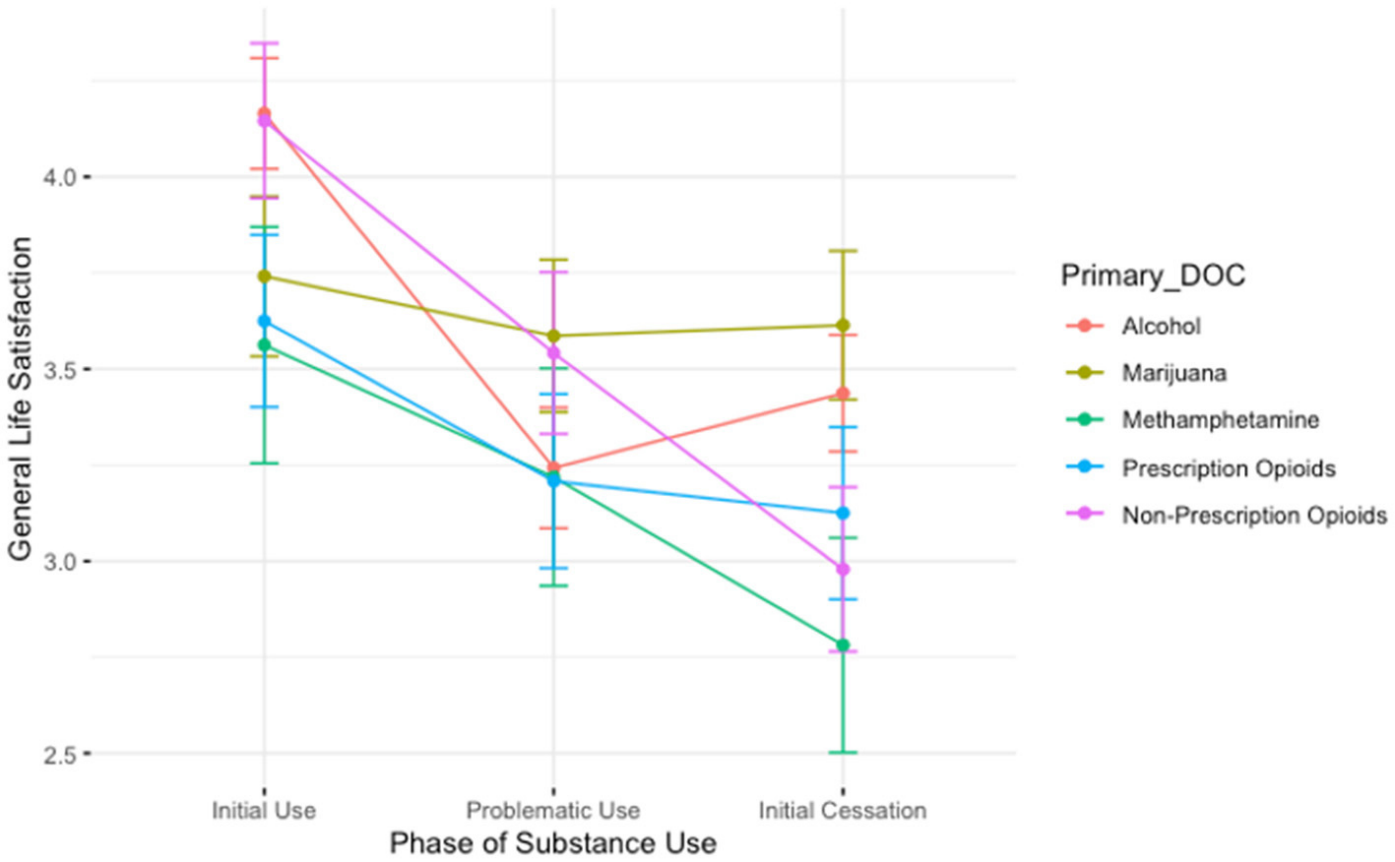
A plot showing the average general life satisfaction scores for participants during the initial stages of use, the height of problematic use, and during the initial stages of cessation. Lines are colored by participant primary substance of choice. Those who used marijuana report higher general life satisfaction than those who used alcohol during the height of problematic use.

The ANOVA evaluating the association between drug of choice and time for the three time-points *during* use (initial use, height of use, and initial cessation) revealed a main effect of time; Figure 6 depicts the results. Relative to the initial phases of substance use, people report lower feelings of general life satisfaction both during the height of problematic use (B = −0.92, *t* = −4.32, *p* < 0.001) and during the initial stages of quitting/reducing use (B = −0.73, *t* = −3.41, *p* < 0.001). We also observed a main effect of drug of choice: relative to those who used alcohol, those who used prescription opioids reported lower life satisfaction (B = −0.54, *t* = −2.02, *p* < 0.05). There was a significant interaction between drug of choice and time: those who used marijuana reported higher general life satisfaction during the height of their problematic use period than those who used alcohol (B = 0.77, *t* = 2.16, *p* < 0.05).

### Current Satisfaction and Well-Being (Post-problem Use)

#### Social Life Satisfaction

We used ANOVA to predict current social life satisfaction by drug of choice (participants with no history of drug use as the reference group) with age, drug use severity, and age of first intoxication as covariates in the model. We observed no significant main effects or interactions in the model; no differences in current social life satisfaction were observed among those who used any drug of choice relative to the comparison group.

#### Romantic Life Satisfaction

We used ANOVA to predict current romantic life satisfaction by drug of choice (participants with no history of drug use as the reference group) with age, drug use severity, and age of first intoxication as covariates in the model. We observed no significant main effects or interactions in the model; however, age was a significant covariate such that for each additional year of age, participants scored 0.03 lower on current romantic life satisfaction (B = 0.03, *t* = −2.66, *p* < 0.01).

#### General Life Satisfaction

We used ANOVA to predict current general life satisfaction by drug of choice (participants with no history of drug use as the reference group) with age, drug use severity, and age of first intoxication as covariates in the model. We observed no significant main effects or interactions in the model.

#### Social Well-Being

We used the validated Social Well-being Scale (range = 7–105) to assess *current* feelings of social well-being; the scale had high internal reliability (Cronbach's alpha = 0.89, CI [0.87, 0.90]). We observed no significant differences between the comparison group of those with no history of problematic drug use and any other drug use class. We observed no significant interaction between drug of choice and time in sobriety, nor any effect of age or drug use severity on current social well-being.

#### Quality of Life

**Figure 7:**
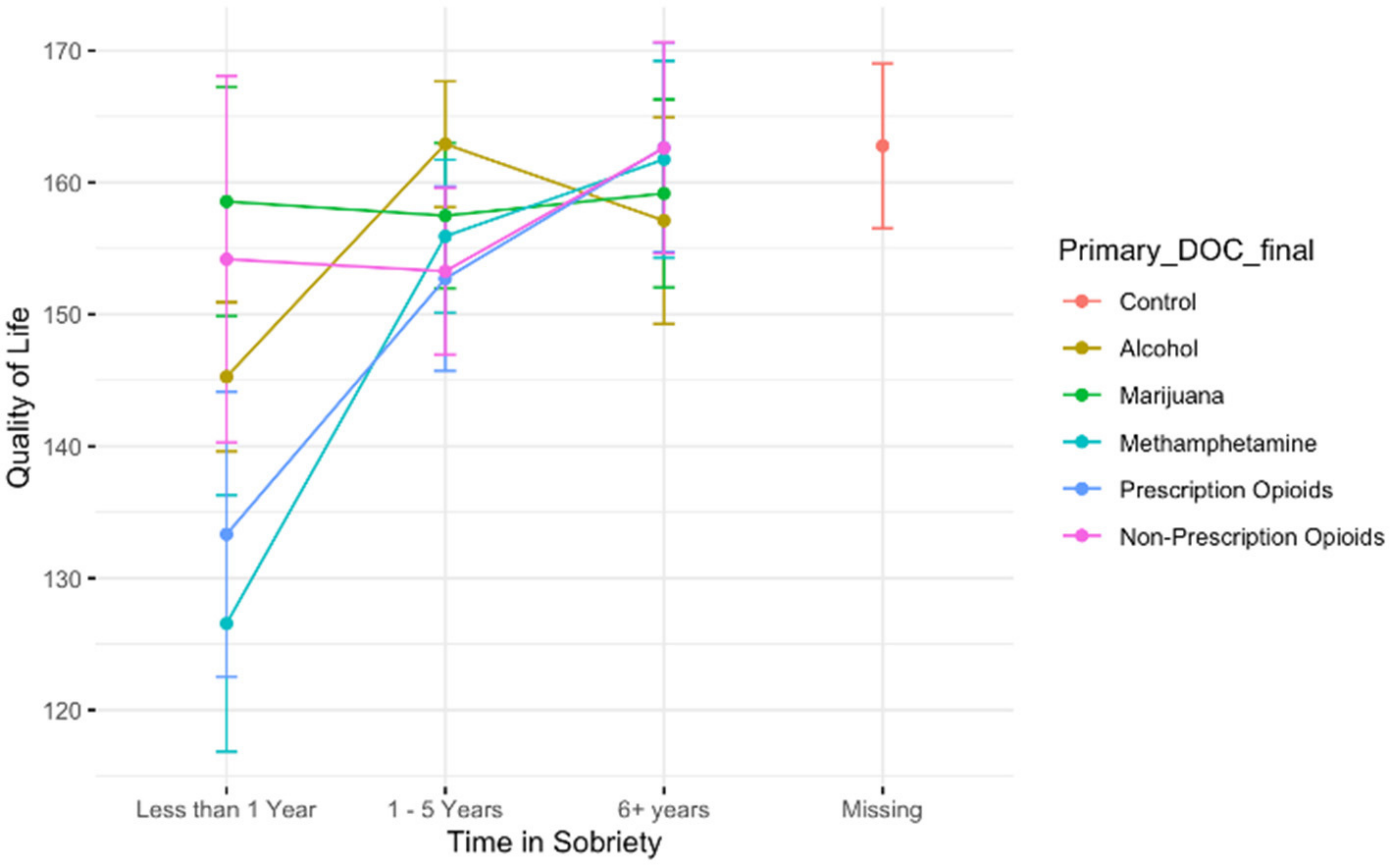
A plot showing the trend that the longer time a person spends in recovery/sobriety, the higher their reported Quality of Life. There is no difference between Quality of Life among the control participants and any of those who have been in sobriety/recovery for six or more years. The highest increases in quality of life from early to later recovery are among those who used methamphetamine or prescription opioids.

The Quality of Life measure (range = 7–245) had high internal reliability (Cronbach's alpha = 0.94, CI [0.94, 0.95]). We observed no significant differences between the comparison group of those with no history of problematic drug use and any other drug use class. However, there is an effect of age, such that for each year older, participants reported a 0.37 higher point quality of life (B = 0.37, *t* = 2.00, *p* < 0.05). Among those who do have a history of problematic substance use, we observed a significant main effect of time in sobriety such that those in the 1–5-year recovery mark report a Quality of Life score that is 15.85 points higher than those with <1 year of recovery time, which is depicted in Figure 7. There is also an effect of drug use severity; for each additional point endorsed on the DAST-10 measure, participants reported a 2.32 lower quality of life score (B = −2.32, *t* = −2.11, *p* < 0.05). We report no significant main effect of drug of choice, and no effect of age. See Figure 7.

## Discussion

The changes in social, romantic, and general life satisfaction across phases of substance use vary by a person's primary drug of choice. The first portion of this paper assessed differences in groups prior to the initiation of substance use. When reflecting on the time before they had ever tried a substance, those who used marijuana report lower social life satisfaction, and those who used prescription opioids report both higher romantic and general life satisfaction.

In the second portion of the paper, we assessed differences that arise during the course of active substance use. Interestingly, the highest reported social, romantic, and general life satisfaction for those who used prescription opioids is *before the initiation of any substance use* at all, whereas the highest reported social, romantic, and general life satisfaction for those who used alcohol, marijuana, methamphetamine, and non-prescription opioids occurs *during the initial stages of substance use*. It may be the case that those who used prescription opioids feel low satisfaction with social, romantic, and/or general life during the course of substance use, and thus report higher levels of pre-substance use satisfaction, because in hindsight that may be the time in which they felt the most satisfied. One possible reason is that a higher proportion of those initiating prescription opioid use may have begun their use due to physical pain: they may think of their time prior to the onset of a medical issue as more satisfactory than the time when they initiated misuse of the prescription—which may have occurred in the context of pain. Thus, there was no “honeymoon” period where the drug effect was highly pleasurable in the initial stages of use. Another plausible explanation for the differences in satisfaction between those who used prescription vs. non-prescription opioids is that those who used prescription opioids tended to report higher levels of socioeconomic well-being relative to their counterparts who used non-prescription opioids. It could be the case that change from pre-substance use to the initial stages of use produced a larger decline in social, romantic, and general life satisfaction among those who used prescription opioids because their lives pre-substance use may have been more socially and economically enriched. Their starting point may have yielded a “farther fall” with more negative consequences experienced as a result of substance use, such as a loss of familial or social ties, or economic loss. Future work should address these issues in a larger sample, and collect data cross-sectionally and prospectively to determine if there are pre-existing differences in feelings of social or romantic connections in the beginning of substance use among those who use different substances that may put people at higher risk of developing a substance use disorder.

Perhaps unsurprisingly, participants across all substance groups report a decline in social, romantic, and general life satisfaction as substance use moves from the initial stages, to the height of problematic use, and into the initial stages of cessation. For social life, those who used prescription opioids and marijuana reported lower satisfaction throughout the course of substance use than their counterparts who used alcohol. The lowest reported social satisfaction was among those who used prescription opioids during the height of their problematic use. For romantic life satisfaction, those who used non-prescription opioids reported the lowest scores, specifically during the period of initial cessation. Interestingly, those who used non-prescription opioids and methamphetamine were the only groups to report a decrease in romantic life satisfaction from the height of problematic use to the initial period of cessation. This is in line with prior work on the role of methamphetamine in romantic and sexual encounters; and is paradoxically also in line with work suggesting that those who use non-prescription opioids report little interest in romantic partners in the midst of their use. It may be that those who used opioids report lower romantic life satisfaction during the initial period of cessation as romantic encounters begin to become appealing once more, but they do not yet have a partner. For general life satisfaction, once again those who used prescription opioids report lower satisfaction than their peers. Compared with feelings of satisfaction before the initiation of substance use, those who used prescription opioids also reported a larger decrease in satisfaction in their social life and general life compared with those who used alcohol, and a larger decrease in their romantic life satisfaction than those who used marijuana or methamphetamine (see Appendix for detailed results on change scores).

In the third and final section of the paper, we addressed differences in current feelings of satisfaction between people with different drugs of choice alongside those who have no history of problematic substance use, and we see a new pattern emerge. There were no significant differences between the comparison group and the participants with a history of problematic substance use in any of the three domains: social, romantic, or general life. Beyond the current single-item measures of life satisfaction, we also asked participants to complete two full scale measures on social well-being and quality of life. Again, we saw that participants with a history of substance use report similar levels of social well-being and quality of life compared with those with no history of substance use. We then separately assessed only those with a history of drug use, including covariates such as age of first intoxication and time in recovery. Those in the 1–5-year period of recovery reported a higher quality of life than those with fewer than 1 year in recovery. Yet, scores among those with over 6 years in recovery match the control group. In short, quality of life is higher among those who are in recovery for longer. Altogether, participants with and without a history of problematic substance use look similar in terms of current social life satisfaction, romantic life satisfaction, social well-being, and quality of life. These findings suggest a hopeful message: although satisfaction across domains of life is low during problem use, it returns to normal with sustained remission. These findings are also important from a clinical and policy perspective. From a clinical perspective, life satisfaction predicts who will remain in recovery: for instance, those who report higher satisfaction in their own lives are more likely to remain in recovery at a 2-year follow-up, even when controlling for motivation and commitment to abstinence (37). Additionally, for medical providers, understanding how substance use impacts general well-being can potentially enhance patient-provider interactions and lead to improvements in substance use disorder/overall well-being screening measures within a healthcare setting. From a policy perspective, policymakers can aim to support and implement programs that are demonstrated to increase quality of life among those who use substances—which is associated with a decreased risk of relapse. For example, policymakers can increase the availability and accessibility of methadone maintenance programs, which have been shown to increase quality of life among those who are in recovery from opioid use (38).

While we aimed to assess differences between those with different primary drugs of choice, we had a specific interest in looking at changes over time in satisfaction among those who used opioids. There is a strong biopsychosocial rationale as to why opioid use could produce divergent social satisfaction changes relative to other drugs. In human romantic relationships, endorphins (a class of endogenous opioid) increase with sexual behavior (39). Behaviorally, opioid use disorder negatively impacts relationships across the board with detrimental outcomes for familial, social, and romantic ties. This disruption is somewhat more complex for romantic partners: individuals who engage in chronic opioid use —males in particular—tend to lose sexual interest in their partners, with impairments in both psychological and physiological arousal (40). Given the centrality of the endogenous opioid system in the experience of social connection (see BOTSA), it has been proposed that problem use of opioids may be more closely linked to social disconnection than problem use of other substances (13). The directionality of the relationship between social isolation and opioid use remains unclear. However, we posit that the relationship is likely bidirectional, such that pre-existing feelings of social exclusion or isolation put a person at higher risk of developing problem opioid use, and that chronic problem opioid use exacerbates the lived experience of social isolation and blunts feelings of reward associated with social connection [see (13)].

We did not, however, have specific hypotheses about differences between those who used prescription and non-prescription opioids in terms of satisfaction or social well-being. Prior work has demonstrated differences in demographic characteristics between those who use prescription opioids relative to non-prescription, and has even reported that those with lower incomes hold fewer stigmatizing attitudes toward people with an opioid use disorder (41). Thus, people who initiate prescription opioid use may belong to social circles where their peers or family members are more likely to socially exclude or stigmatize them for their substance use. Additionally, people who report prescription opioid use may feel shame about misusing a prescribed medication intended for therapeutic purposes (42), which is unlikely to be a factor with other substances observed. Another variable that may explain differences between the groups is the legality of a person's drug of choice: alcohol is legal throughout the United States, marijuana is legal in several states (although the sample is of people who *formerly* used marijuana and it may not have been legal at the time of their use), and prescription opioid use is sometimes initiated under the legal supervision of a healthcare professional. However, methamphetamine and non-prescription opioids are both illicit substances in the United States. People who reported methamphetamine or non-prescription opioids as their drug of choice may have different experiences than those who are using more licit and less stigmatized substances. Future work should aim to address the differences between satisfaction in life, including the social domain, among those who use prescription and non-prescription opioids, as well as among those who interact with the criminal justice system during the course of their substance use.

This study is not without limitations. First, our data is retrospective data, and in addition to forgetting, people often interpret their past according to narratives (such as redemption narratives) which may affect the feelings they ascribe to their past selves over time (43). Secondly, this sample is comprised of adults in the United States, and future work should address how these patterns may differ in adolescent and young adult populations both in the United States and abroad. Adolescent opioid use differs from adult opioid use in several ways: (1) developmentally, adolescents show increased reward sensitivity to opioids relative to adults' reward sensitivity (44), and (2) the onset of adolescent opioid use has been linked to structural factors, such as parental opioid use and medical treatment for injuries [such as through sports; (45, 46)]. Thus, exploring satisfaction in life domains related to adolescent substance use, particularly with opioid use, is a worthwhile preventative public health endeavor. Third, using substance of choice as a variable does not allow us to make inferences about the unique effects of polysubstance use, which many individuals in our sample likely engaged in throughout the course of their substance use. Specifically, the majority of people who use opioids use a combination of substances, and may use both prescription and non-prescription opioids—preference for one over the other does not equate to exclusive use of the preferred substance (47, 48). Fourth, we do not have a large enough sample size to make statistical comparisons between the two subgroups of our sample in recovery with a history of problematic drug use: those who are abstinent, and those who engage in casual drug use. Finally, as our sample was predominantly white, we are unable to comment on the potential role of race or ethnicity. Prior work using the framework of minority stress theory has reported that men who identify as a racial and sexual minority are more likely to engage in substance use behaviors as a form of avoidant coping for social stress and discrimination (49).

There are also several strengths to the current study. The present study is the first to our knowledge to chart the time-course of satisfaction with social life, romantic life, and general life satisfaction among people with a history of problematic substance use—broken down by primary drug of choice. In order to effectively treat those with substance use disorders, we must understand in which life domains people are suffering. This work paints a picture that those who formerly used prescription opioids experience larger declines across several domains of life satisfaction throughout the course of their substance use: social life, romantic life, and general life. Secondly, this decline in satisfaction among those who used prescription opioids is between the time before substance use initiation to the time when they had just initiated substance use, whereas those who used other substances reported increases in life satisfaction domains from pre-substance use to initial phases, and declines following that. Feeling connected and satisfied in life domains is important for overall well-being and longevity, and is particularly important for those suffering from prescription opioid use disorders across the span of their substance use and into recovery.

## Data Availability Statement

The raw data supporting the conclusions of this article will be made available by the authors, without undue reservation.

## Ethics Statement

The studies involving human participants were reviewed and approved by University of Southern California Institutional Review Board. Written informed consent for participation was not required for this study in accordance with the national legislation and the institutional requirements.

## Author Contributions

NC created the project idea in collaboration with JM and NC completed the outline of the manuscript, and the analyses. PM contributed to the literature review and writing the introduction. VV also contributed to the literature review and writing the introduction. JM was the supervising author, consulting on data analyses, presentation, and authorship. All authors contributed to the article and approved the submitted version.

## Funding

The University of Southern California Department of Psychology awarded graduate student researchers with research funds through a department grant. These funds were used for participant payments on Prolifiic.co.

## Conflict of Interest

The authors declare that the research was conducted in the absence of any commercial or financial relationships that could be construed as a potential conflict of interest.

## Publisher's Note

All claims expressed in this article are solely those of the authors and do not necessarily represent those of their affiliated organizations, or those of the publisher, the editors and the reviewers. Any product that may be evaluated in this article, or claim that may be made by its manufacturer, is not guaranteed or endorsed by the publisher.
