# Supplementary material for: Changes in Social, Romantic, and General Life Satisfaction Over the Course of a Substance Use Disorder
Source: Front Psychiatry. 2021 Oct 28;12:734352. doi: 10.3389/fpsyt.2021.734352 (PMC8581205; doi:10.3389/fpsyt.2021.734352)
Supplement: Supplementary file 1 [file Presentation_1.pdf]

APPENDIX

Statistical Model Formulas in R:

Prior to Substance Use

aov([Social OR Romantic OR General Life] Satisfaction ~ Drug of Choice + Substance Use Severity + Current Age + Age of First Intoxication)

During the Course of Substance Use

Model 1: aov([Social OR Romantic OR General Life] Satisfaction ~ Drug of Choice*Time)

Model 2: aov(Change from Pre-Substance [Social OR Romantic OR General Life] Satisfaction ~ Drug of Choice*Time)

Model 3: aov(Residuals of change scores from Pre-Substance [Social OR Romantic OR General Life] Satisfaction ~ Drug of Choice*Time)

Current Satisfaction (Post-Problem Use)

[Social OR Romantic OR General Life] Satisfaction ~ Drug of Choice + Age + Age of First Intoxication + Substance Use Severity

Current Wellbeing and Quality of Life (Post-Problem Use)

Model 1:[Social Wellbeing OR Quality of Life] ~ Drug of Choice + Age

Model 2: [Social Wellbeing OR Quality of Life] ~ (Time in Sobriety*Drug of Choice) + Age + Substance Use Severity

Secondary Analyses

Social Life Satisfaction: Change Scores

We observed a significant main effect of time: relative to the initial phases of substance use, people report a larger reduction in social life satisfaction from pre-substance use times to the problematic use stage ($B = -0.70$, $t = -2.50$, $p < 0.05$) and to the initial stages of quitting/reducing

use (B = -0.69, t = -2.46, p < 0.05). We also observed a significant main effect of substance: relative to those who use alcohol, those who use prescription opioids report a significantly larger reduction in social life satisfaction from pre-substance use levels to during substance use levels (B = -1.21, t = -3.45, p < 0.001).

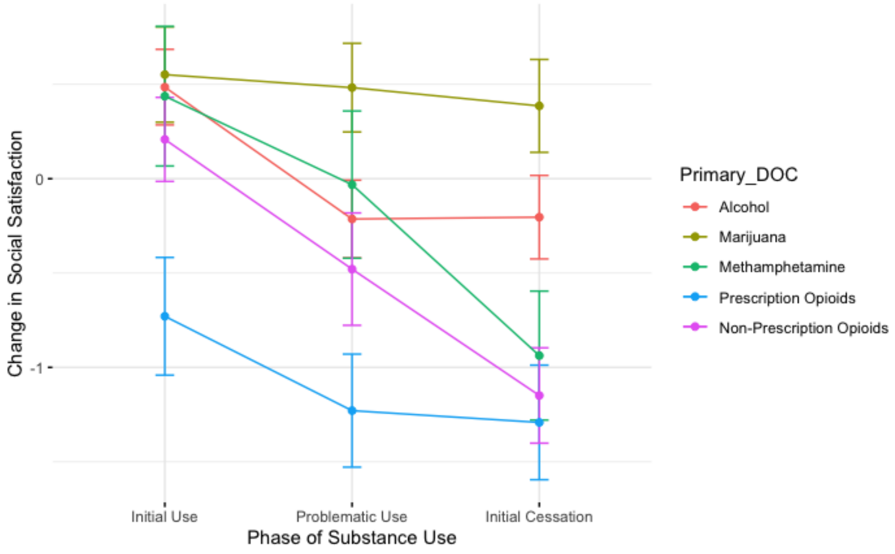

Appendix Figure 1: Change scores in social life satisfaction relative to the period of time before substance use.

Romantic Life Satisfaction: Change Scores

We observed a significant main effect of time: relative to the initial phases of substance use, people report a larger reduction in romantic life satisfaction from pre-substance use times to the problematic use stage (B = -0.58, t = -1.99, p < 0.05). There was no main effect of substance nor interaction between time and substance of choice.

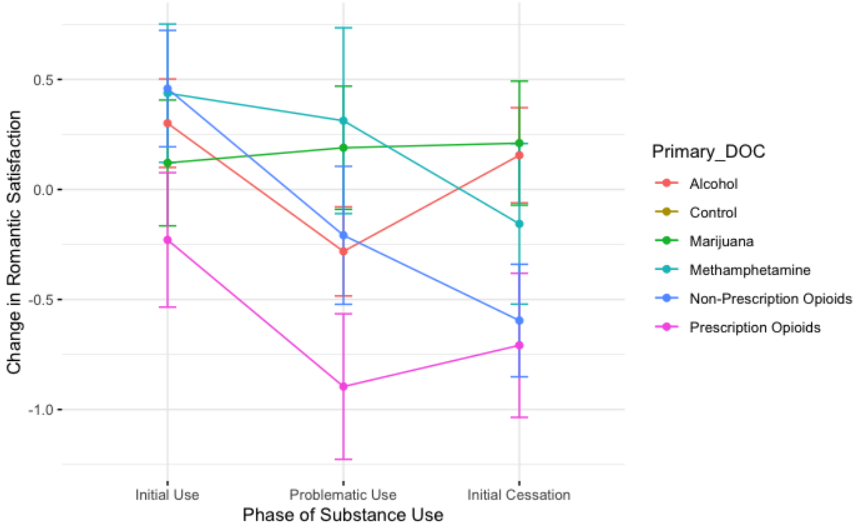

Appendix Figure 2: Change scores in romantic life satisfaction relative to the period of time before substance use.

General Life Satisfaction: Change Scores

We observed a significant main effect of time: relative to the initial phases of substance use, people report a larger reduction in general life satisfaction from pre-substance use times to the problematic use stage (B = -0.92, t = -3.56, $p < 0.001$) and to the initial stages of quitting/reducing use (B = -0.73, t = -2.81, $p < 0.01$). We also observed a significant main effect of substance: relative to those who use alcohol, those who use prescription opioids (B = -1.27, t = -3.91, $p < 0.001$) and those who use methamphetamine (B = -0.75, t = -1.99, $p < 0.05$) report a significantly larger reduction in general life satisfaction from pre-substance use levels to during substance use levels.

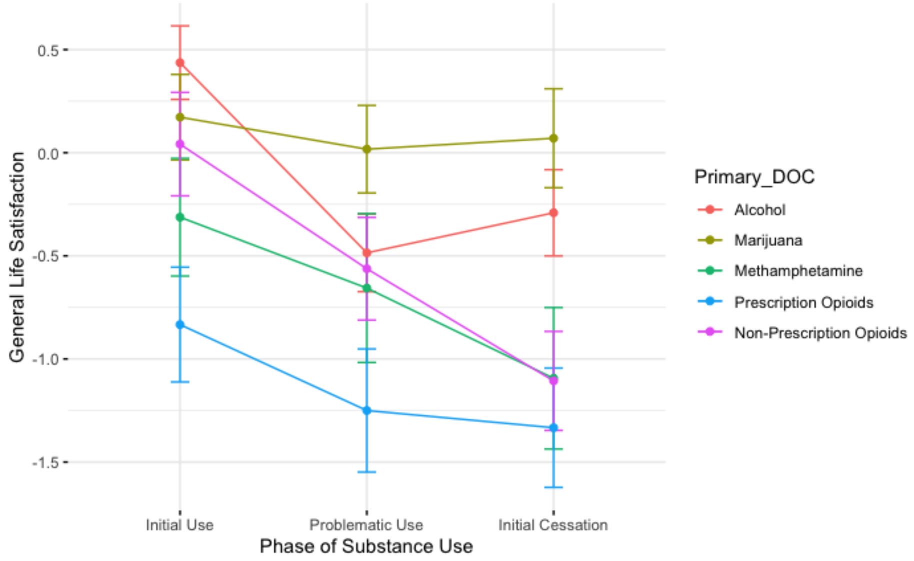

Appendix Figure 3: Change scores in general life satisfaction relative to the period of time before substance use.

Social Life Satisfaction: Residual Change Scores

This model is the residuals of the change scores (which measure change from pre-substance use to each of the phases of active substance use) regressing out age and substance use severity. We observed no main effects of time. We did observe a main effect of drug of choice: relative to those who use alcohol, those who use prescription opioids report lower social life satisfaction across the active phases of drug use ($B = -0.58$, $t = -3.39$, $p < 0.001$).

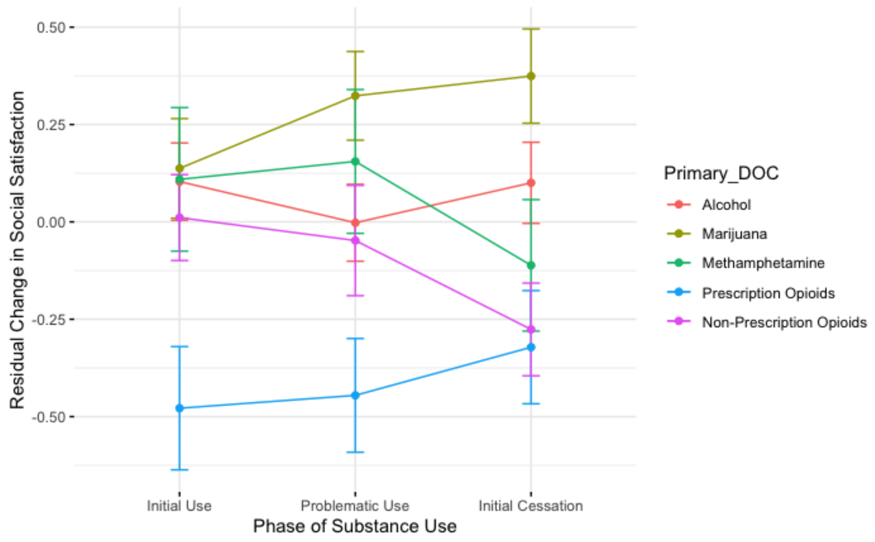

Appendix Figure 4: Residual change scores in social life satisfaction relative to the period of time before substance use.

Romantic Life Satisfaction: Residual Change Scores

This model is the residuals of the change scores (which measure change from pre-substance use to each of the phases of active substance use) regressing out age and substance use severity. We observed no main effects of time. We did observe a main effect of drug of choice: the ANOVA indicated a main effect between drug classes. The difference was not between a specific drug class and alcohol (the reference), thus we performed a post-hoc Tukey test which showed that those who use prescription opioids report lower romantic life satisfaction than those who use marijuana (mean difference = -0.34, 95% CI [-0.65, -0.03], $p < 0.05$) and those who use methamphetamine (mean difference = -0.40, 95% CI [-0.76, -0.04], $p < 0.05$). There are no significant interactions between time and drug of choice.

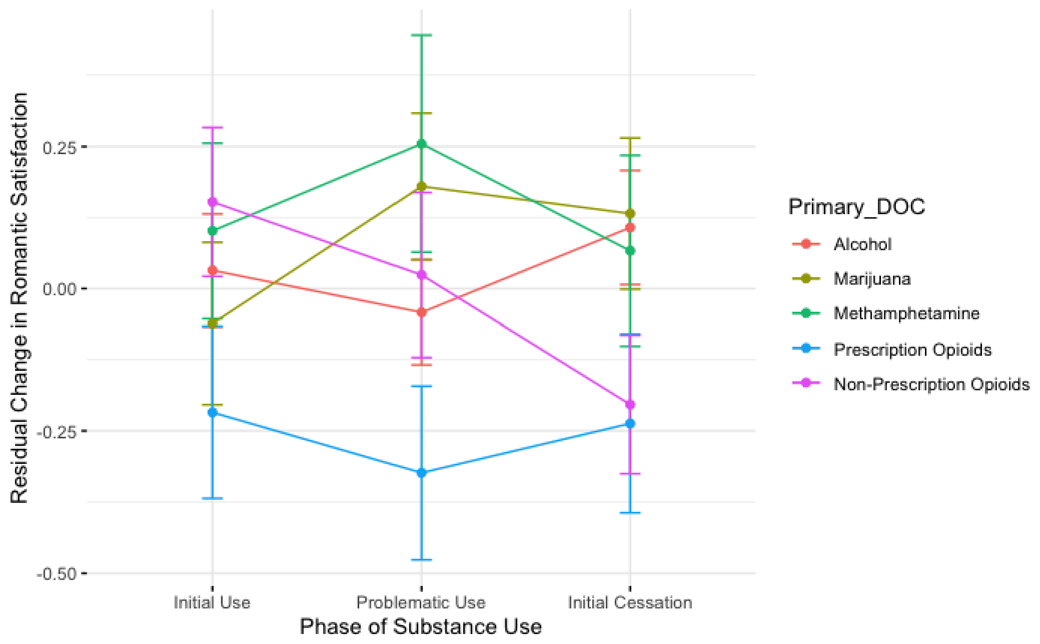

Appendix Figure 5: Residual change scores in romantic life satisfaction relative to the period of time before substance use.

General Life Satisfaction: Residual Change Scores

This model is the residuals of the change scores (which measure change from pre-substance use to each of the phases of active substance use) regressing out age and substance use severity. We observed no main effects of time. We did observe a main effect of drug of choice: relative to those who use alcohol, those who use prescription opioids (B = -0.68, t = -3.97, $p < 0.001$) and those who use methamphetamine (B = -0.39, t = -1.96, $p < 0.05$) report lower general life satisfaction across the active phases of drug use.

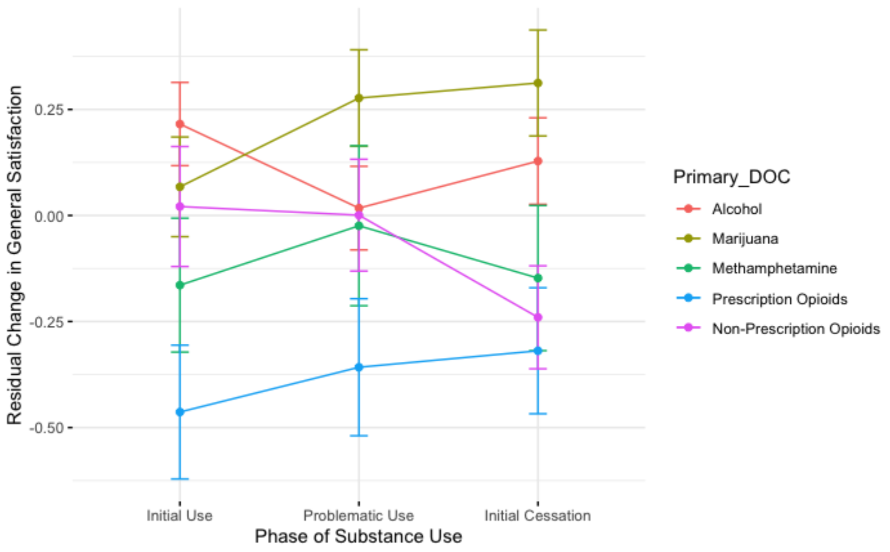

Appendix Figure 5: Residual change scores in general life satisfaction relative to the period of time before substance use.
